# A comparative transcriptome analysis of the novel obligate methanotroph *Methylomonas* sp. DH-1 reveals key differences in transcriptional responses in C1 and secondary metabolite pathways during growth on methane and methanol

**DOI:** 10.1186/s12864-019-5487-6

**Published:** 2019-02-12

**Authors:** Anh Duc Nguyen, Donghyuk Kim, Eun Yeol Lee

**Affiliations:** 1 Department of Chemical Engineering, Kyung Hee University, Yongin, 17104 Republic of Korea; 2 School of Energy and Chemical Engineering & School of Biological Sciences, Ulsan National Institute of Science and Technology (UNIST), Ulsan, 44919 Republic of Korea

**Keywords:** Methanotroph, Serine cycle, RuMP cycle, TCA, Carotenoid biosynthesis pathway, Hopanoid biosynthesis pathway, RNA-seq

## Abstract

**Background:**

Methanotrophs play an important role in biotechnological applications, with their ability to utilize single carbon (C1) feedstock such as methane and methanol to produce a range of high-value compounds. A newly isolated obligate methanotroph strain, *Methylomonas* sp. DH-1, became a platform strain for biotechnological applications because it has proven capable of producing chemicals, fuels, and secondary metabolites from methane and methanol. In this study, transcriptome analysis with RNA-seq was used to investigate the transcriptional change of *Methylomonas* sp. DH-1 on methane and methanol. This was done to improve knowledge about C1 assimilation and secondary metabolite pathways in this promising, but under-characterized, methane-bioconversion strain.

**Results:**

We integrated genomic and transcriptomic analysis of the newly isolated *Methylomonas* sp. DH-1 grown on methane and methanol. Detailed transcriptomic analysis indicated that (i) *Methylomonas* sp. DH-1 possesses the ribulose monophosphate (RuMP) cycle and the Embden–Meyerhof–Parnas (EMP) pathway, which can serve as main pathways for C1 assimilation, (ii) the existence and the expression of a complete serine cycle and a complete tricarboxylic acid (TCA) cycle might contribute to methane conversion and energy production, and (iii) the highly active endogenous plasmid pDH1 may code for essential metabolic processes. Comparative transcriptomic analysis on methane and methanol as a sole carbon source revealed different transcriptional responses of *Methylomonas* sp. DH-1, especially in C1 assimilation, secondary metabolite pathways, and oxidative stress. Especially, these results suggest a shift of central metabolism when substrate changed from methane to methanol in which formaldehyde oxidation pathway and serine cycle carried more flux to produce acetyl-coA and NADH. Meanwhile, downregulation of TCA cycle when grown on methanol may suggest a shift of its main function is to provide de novo biosynthesis, but not produce NADH.

**Conclusions:**

This study provides insights into the transcriptomic profile of *Methylomonas* sp. DH-1 grown on major carbon sources for C1 assimilation, providing in-depth knowledge on the metabolic pathways of this strain. These observations and analyses can contribute to future metabolic engineering with the newly isolated, yet under-characterized, *Methylomonas* sp. DH-1 to enhance its biochemical application in relevant industries.

**Electronic supplementary material:**

The online version of this article (10.1186/s12864-019-5487-6) contains supplementary material, which is available to authorized users.

## Background

Single-carbon (C1) feedstock such as methane and methanol has great potential for a number of applications and has shown flexibility when used with biocatalysts and in bioconversion processes to produce various products [1, 2]. Methane is the main component of natural (and shale) gas and biogas. Recently, expansion of the global methane market has improved accessibility, resulting in decreasing gas prices. This has made methane an attractive next-generation feedstock [3]. Generally, chemical conversions of methane to other chemicals require a high input of energy because of the high activation energy of the C-H bond [4]. Thus, biological conversions of methane to higher value products using methanotrophs has grown more attractive recently. As intermediates in the aerobic methane oxidation process, methanotrophs are capable of utilizing methanol as a sole carbon source and utilize a similar pathway to that for methane assimilation [2]. Methanol is also an attractive carbon substrate because of its high production volume and low market price [5]. A wide range of methylotrophic bacteria has been used to produce multiple chemically valuable compounds, including single-cell proteins, biopolymers, lipids, methanol, ectoine, and vitamin B12 [6]. In addition, genetically engineered methanotrophs have served as promising and powerful production platforms to over-produce several non-native compounds such as carotenoids, lactic acid, succinic acid, 1,4-butanediol and 2,3-butanediol [7–9]. In an effort to expand on the collection of bacterial bioconversion platforms, a novel obligate methanotroph strain, *Methylomonas* sp. DH-1, was recently isolated from the sludge of a brewery. However, the strain had yet to be characterized for its bioconversion abilities with molecular-level experiments [10]. *Methylomonas* sp. DH-1 showed highly efficient bioconversion of methane to methanol, with a titer of 1.34 g per liter. This is more efficient than the conversion rate of previously reported biocatalysts [10]. Furthermore, the strain’s ability to tolerate high concentrations of methanol (up to 7% [*v*/v]) offers advantages for high-titer methanol production [10]. Recently, *Methylomonas* sp. DH-1 also proved to be a novel and competent biocatalyst for the conversion of propane to acetone, with a titer of 16.62 mM under mild reaction conditions [11]. Furthermore, *Methylomonas* sp. DH-1 may become a biotechnologically important biocatalyst for its ability to produce several carotenoids (unpublished report).

Development of next-generation sequencing technology enabled the sequencing of genomes of several methanotrophs, and the genomes of those methanotrophs provided essential information for the reconstruction of methane metabolism in methanotrophs [12, 13]. In addition, multi-omics studies (which integrate transcriptomics, metabolomics, proteomics, and genomics) have provided insights to assess various metabolic engineering targets for methanotrophs [8]. Indeed, several previous studies using a multi-omics approach to analyze multiple model methanotrophs have been reported [14–18]. Recently, in our previous work, a complete genome sequence for *Methylomonas* sp. DH-1 was determined [19]. The strain contains one 4.86 Mb chromosome and one 278 kb plasmid, pDH1 [19]. The availability of the complete genome sequence of *Methylomonas* sp. DH-1 provided an essential background for revisiting a genome-based reconstruction of methane metabolism. But to date, a comprehensive transcriptome analysis of *Methylomonas* sp. DH-1 is still elusive, and mechanisms responsible for the methanol tolerance of *Methylomonas* sp. DH-1 have yet to be investigated. An RNA-sequencing approach has been used to investigate the transcriptome and has provided insights into the methane metabolism of type I [16–18] and type II [14, 15] methanotrophs. In this study, we first detail the genome-wide transcriptional responses of methane metabolism and secondary metabolite production in *Methylomonas* sp. DH-1 during growth on methane. We then offer a comparative transcriptomic analysis conducted with cells grown on methane and methanol. This analysis revealed differences in the transcriptional response of multiple metabolic pathways which are relevant to C1 assimilation, secondary metabolite production, and oxidative stress.

## Methods

### Bacterial growth conditions

*Methylomonas* sp. DH-1 was isolated from the activated sludge of a brewery plant based on NMS medium using enrichment culture with methane as sole carbon source as described in our previous work [10]. Liquid pre-cultures were grown in 180 ml baffled-flask with 10 ml NMS with a supplement of 30% methane (*v*/v) as a sole carbon source at 30 °C and 230 rpm, sealed with a screw cap. The precultures were then inoculated in a 500 ml baffled flask sealed with a screw cap containing 50 ml of NMS and methane also was supplied to a final concentration of 30% (*v*/v) by gas substitution using a gas-tight syringe, and the headspace was refreshed every day. The methanol-cultured *Methylomonas* sp. DH-1 was cultured in the same medium containing 1% (v/v) methanol without added methane. All cultures were grown in triplicate for subsequent RNA extraction and sequencing.

### Total RNA isolation and sequencing

For sequencing library preparation, 5 ml of microbial culture broths containing either methane- or methanol-grown cultures in mid-exponential phase were harvested, and total RNA was stabilized using the bacterial reagent RNAprotect (Qiagen, Hilden, Germany). Cells were lysed and total RNA was extracted using a GeneJET RNA Purification Kit (Thermo Fisher Scientific, USA), following the manufacturer’s protocol. Total RNA quality and quantity were measured using an RNA 6000 Nano kit with the Agilent Bioanalyzer (Agilent Technologies, CA, USA). Next, rRNA was removed using the Ribo-Zero rRNA removal kit for gram-negative bacteria (Epicentre, Madison, WI, USA), and the remaining RNA was used for creating the sequencing library using the TruSeq Stranded Total RNA Sample Prep Kit (Illumina, USA) according to the manufacturer’s instructions. Transcriptome sequencing was conducted using the Illumina/Hiseq-2000 RNA sequencing platform (Macrogen, Korea).

### Quantification of differentially expressed genes

After evaluating the quality of the raw sequence data with FastQC (http://www.bioinformatics.babraham.ac.uk/projects/fastqc/), further quantitative analyses were performed. Illumina sequencing (of triplicates) was aligned against the genome sequence of *Methylomonas* sp. DH-1 (NZ_CP014360 for the chromosome and NZ_CP014361 for the plasmid). The Bowtie tool was used with a maximum insert size of 1000 bp and with two maximum mismatches after trimming 3 bp at the 3′ ends under the default options. The sequence alignment/map (SAM) files were post-processed using SAMTools (http://samtools.sourceforge.net/), by first converting to binary alignment/map (BAM) files and then sorting and indexing those BAM files. Indexed and sorted BAM files generated from SAMTools were analyzed by Cufflinks and Cuffdiff to calculate values for fragments per kilobase of exon per million fragments (FPKM) and differential expression, respectively (with default options and a library type of dUTP RNA-seq). Genes from the Cuffdiff output showing differential expression with a log2 fold change ≥1.0 and a false discovery rate (FDR) value ≤0.01 were considered as differentially expressed genes in our study.

### Clusters of orthologous groups (COGs) functional assignment and ortholog calculation

All of the CDS regions were assigned to different functional categories based on the Clusters of orthologous genes (COGs) designation [20]. The ortholog calculation was performed using InParanoid software [21].

## Results

### Genome-wide transcriptome profiling

Gene expression analysis was carried out on *Methylomonas* sp. DH-1 grown in NMS medium with supplementation of methane or methanol as a sole carbon and energy source. All of the experiments were performed using biological triplicates. RNA-seq was performed as described in the Materials and Methods section. On average, 30 million reads were generated per sample, with a Q30 value higher than 96%, which is considered to be large enough for differential expression analysis in bacteria [22]. The Bowtie algorithm was used for sequence read alignment onto the *Methylomonas* sp. DH-1 reference sequence (NZ_CP014360 and NZ_CP014361). On average, 98% of reads were mapped onto the *Methylomonas* sp. DH-1 reference genome. The Bowtie was run with the following options: a maximum insert size of 1000 bp and 2 maximum mismatches after trimming 3 bp at the 3′ ends, with default parameters for the other options. The relative expression level, generated as FPKM values, was calculated to compare gene expression levels within and across biological replicates. A total of 4318 CDS regions in the chromosome were analyzed, while 129 CDS regions without sufficient alignments were removed from further analysis. For the plasmid, 7 among 242 CDS regions were excluded because the number of mapped reads was low. Using the calculated relative expression levels, genes were grouped into 6 expression categories (omitting rRNA genes) following the method by Vorobev et al. [15]: very high (500 FPKM), high (500 to 200 FPKM), intermediate (200 to 50 FPKM), low (50 to 10 FPKM), very low (10 to 2 FPKM), and not expressed (below 2 FPKM) (Table 1). The majority of expression levels fell into the *intermediate/low* category, covering 72.64% of genes from methanol-grown culture and 71.83% of genes from methane-grown culture. A small proportion of genes showed *very high/high* expression, covering 6.83 and 5.57% of genes in methane and methanol, respectively (Table 1). Interestingly, most of the genes in the endogenous plasmid (90% in methane- and 87.6% in methanol-grown cultures) showed very high expression (Table 1). Differential expression analysis of *Methylomonas* sp. DH-1 grown on methanol and methane indicated that 261 and 723 genes were upregulated and downregulated, respectively, with a fold-change ≥2 and *P* ≤ 0.05. In the ten most highly expressed genes from cultures grown in methane and methanol, there were two genes for non-coding RNA (ncRNA), one gene encoding transfer-messenger RNA (tmRNA), 3 genes encoding the particulate methane monooxygenase (*pmo*) operon, and four genes encoding hypothetical proteins (Additional file 1: Table S1). Genome analysis indicated that *Methylomonas* sp. DH-1 harbors RNase P, three ncRNAs (RNA component class A [*rnpB*], 6S RNA [*ssrS*], and signal recognition particle sRNA [small type, *ffs*]), and one tmRNA (*ssrA*). Among the three ncRNAs, *rnpB*, which is an essential and ubiquitous ribozyme responsible for the maturation of tRNA [23], was the most highly expressed in *Methylomonas* sp. DH-1, followed by *ssrS* (with the third highest expression in this strain). *ssrA* encodes a unique tmRNA that showed the second highest expression in *Methylomonas* sp. DH-1. Furthermore, expression levels of *ssrS*, which typically interacts with the primary holoenzyme form of RNA polymerase and functions as a global regulator that downregulates transcription for modulating stress and optimizing survival during nutrient limitation [24], were strongly downregulated under methanol growth, suggesting that methanol can be a stress factor affecting the growth of *Methylomonas* sp. DH-1. It was speculated that *ncRNA* (*ssrS* and *rnpB*) and *tmRNA* might serve important roles in the gene regulation of *Methylomonas* sp. DH-1. Additionally, from transcriptional profiling analysis, 1482 genes coding for hypothetical proteins were expressed. Among these genes, 85 showed very high expression levels. These findings suggest that unknown, functional proteins might be important in the metabolism of *Methylomonas* sp. DH-1, and that the functional annotation of these hypothetical proteins needs to be performed.

**Table 1:** Categorization of gene expression level

Description of expression level	FPKM range	Methane (Chromosome)		Methanol (Chromosome)		Methane (Plasmid)		Methanol (Plasmid)	
		Number of genes	% genes	Number of genes	% genes	Number of genes	% genes	Number of genes	% genes
Very high	> 500	286	6.83	233	5.57	218	90	212	87.6
High	200–500	405	9.68	379	9.06	18	7.5	24	9.9
Intermediate	50–200	1609	38.45	1355	32.38	0	0	0	0
Low	10–50	1431	34.19	1651	39.45	0	0	0	0
Very low	2–10	416	9.94	498	11.9	0	0	0	0
Not expressed	< 2	38	0.91	69	1.65	6	2.5	6	2.5

### Expression of genes involved methane oxidation of *Methylomonas* sp. DH-1

**Fig. 1:**
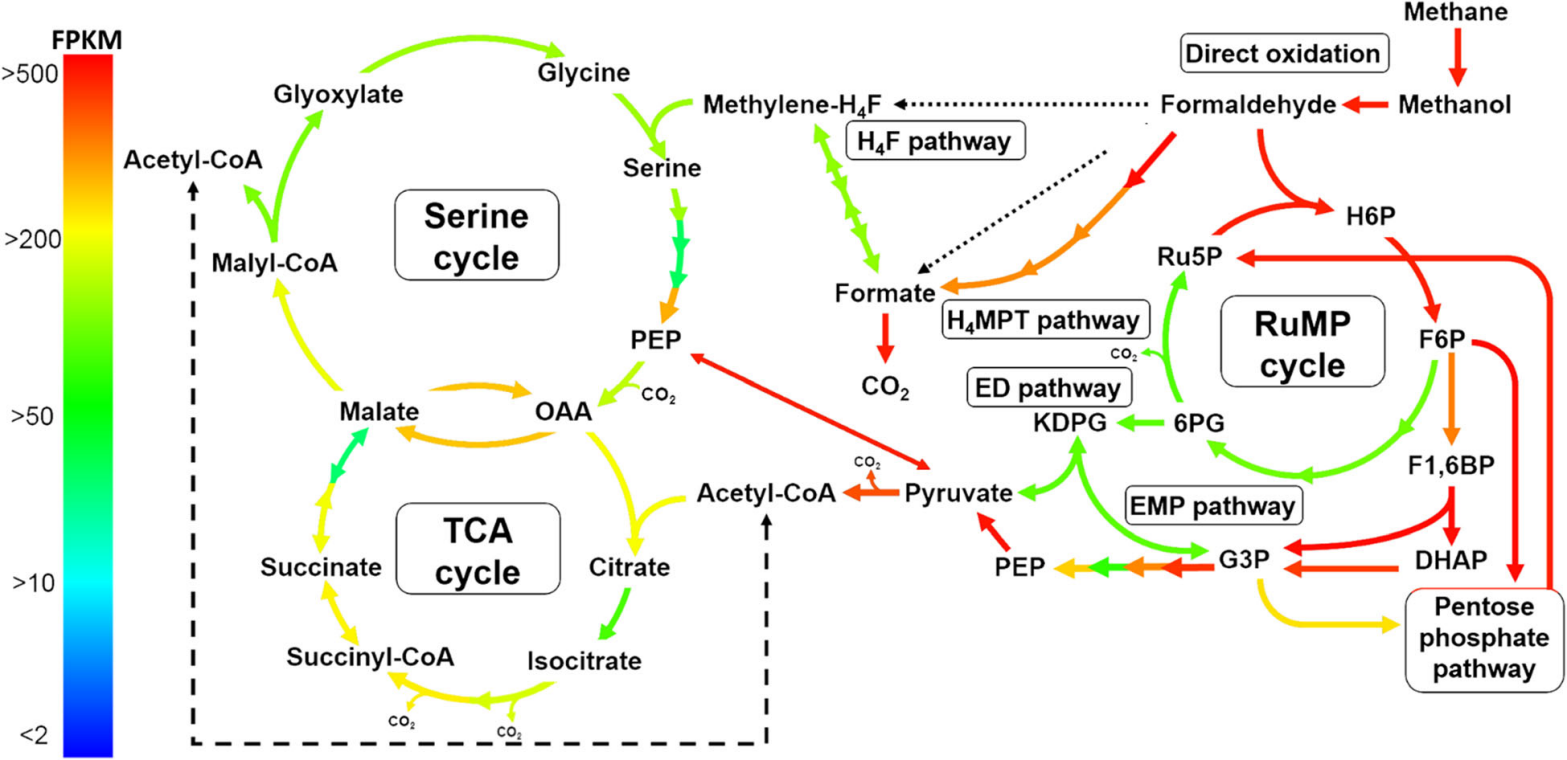
Overview of the central metabolic pathways in *Methylomonas* sp. DH-1 predicted from the genomic annotation and the transcriptomic data mapping. The colors indicate the relative gene expression level. Ru5P: ribulose 5-phosphate, H6P: hexulose 6-phosphate, F6P: fructose 6-phosphate, KDPG: 2-keto-3-deoxy 6-phosphogluconate, F1,6BP: fructose 1,6-bisphosphate, DHAP: dihydroxyacetone phosphate, G3P: glyceraldehyde 3-phosphate, PEP: phosphoenolpyruvate, OAA: oxaloacetic acid

**Fig. 2:**
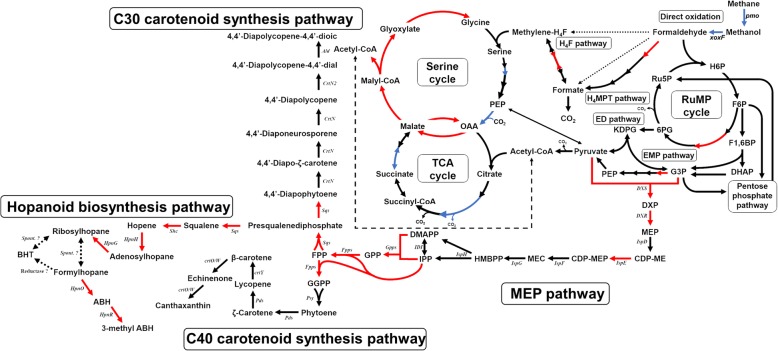
The central metabolism and the secondary metabolites of *Methylomonas* sp. DH-1 grown on methane or methanol as a sole carbon/energy source. Genes highlighted in red and blue were significantly upregulated and downregulated (respectively) on methanol

**Fig. 3:**
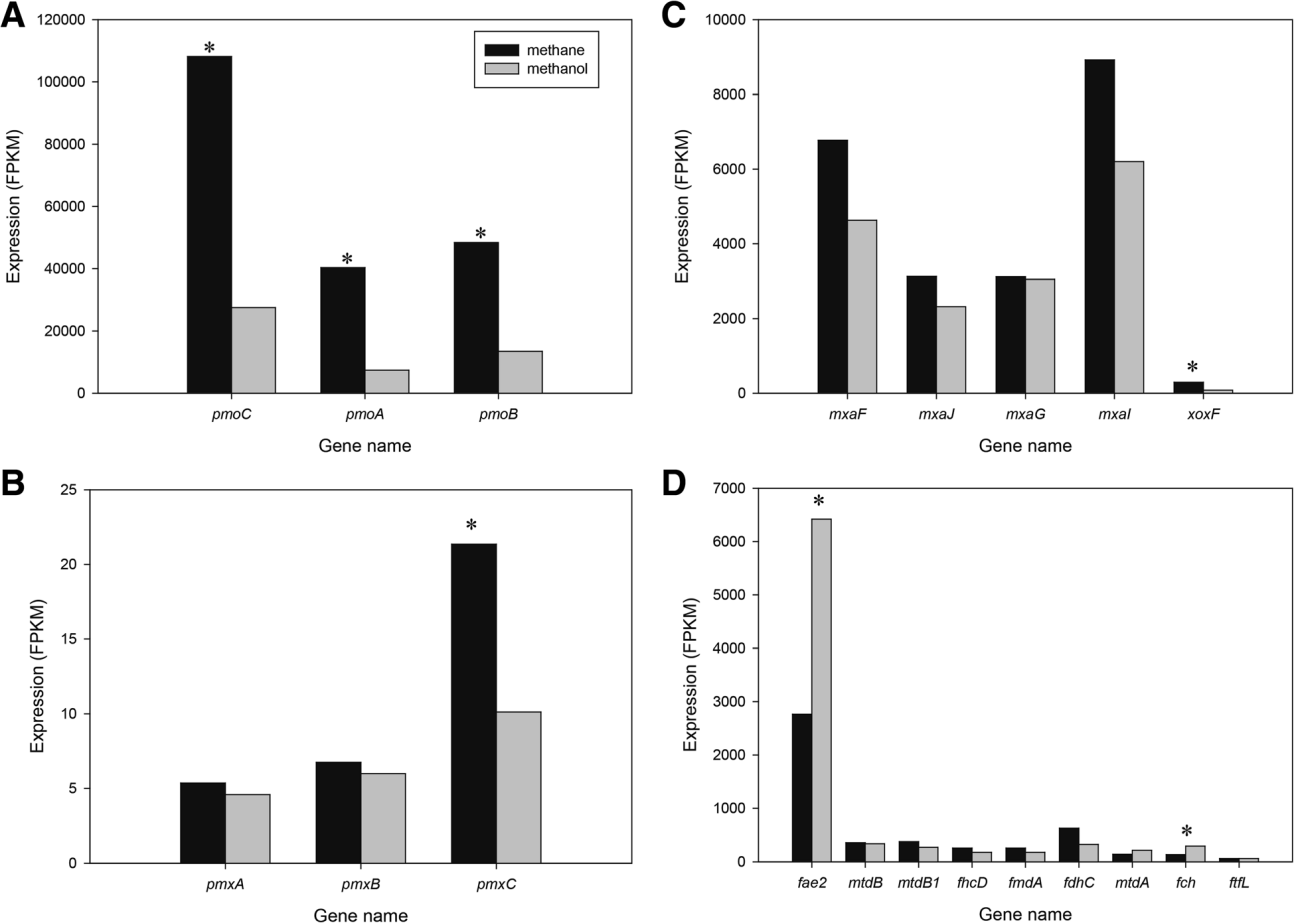
Differential expression of genes involved in C1 metabolism: **a** methane oxidation, **b** non-canonical form *pxmABC*, **c** methanol oxidation, **d** formaldehyde oxidation in *Methylomonas* sp. DH-1 grown on methane (black) and methanol (gray). * Significantly different expression of genes between methane- and methanol-grown cultures (*P* < 0.05)

An overview of the methane metabolism of *Methylomonas* sp. DH-1 grown on methane is summarized in Fig. 1. The relative expressions of genes (FPKM values) are shown in the additional files for growth on methane or methanol. Since *Methylomonas* sp. DH-1 has pathways for C1 assimilation, it was postulated that the genes involved in C1 assimilation would be highly expressed when grown on methane or methanol. As postulated, genes in the pathways for methane or methanol oxidation were highly expressed or very highly expressed. Compared to the C1 metabolic genes in typical obligate methanotrophs, the *Methylomonas* DH-1 genome harbors one copy of the particulate methane monooxygenase (*pmo*) gene cluster and does not contain genes encoding soluble methane monooxygenase (*smo*). The *pmo* gene cluster was the most highly expressed when grown on methane. Among the three genes in the *pmoCAB* gene cluster, which was also very highly expressed, expression of *pmoC* was approximately 2.5-fold higher than the other genes in the same operon (Fig. 3a and Additional file 2: Table S2). Despite *pmo* genes being part of a canonical operon, the transcript abundance of *pmoC* was higher than that of *pmoA* and *pmo*B. This is consistent with previous findings in the alpha-proteobacterial methanotrophs *Methylosinus trichosporium* OB3b [14] and *Methylocystis* sp. strain SB2 [15], and with previous findings in the gamma-proteobacterial methanotrophs *Methylomicrobium alcaliphilum* 20Z [16] and *Methylomicrobium buryatense* 5GB1 [17]. In addition, a sequence-divergent particulate monooxygenase that grouped with the non-canonical form *pmxABC* was found in the *Methylomonas* sp. DH-1 genome, similar to findings in other species from the genera *Methylomonas*, *Methylobacter*, and *Methylomicrobium* [25]. In contrast to the *pmo* operon, the expression of *pmxABC* was very low (Additional file 2: Table S2). When grown on methanol, the expression level of the *pmoCAB* operon was dramatically downregulated, with 2.87, 5.46, and 2.74-fold changes observed respectively for each gene (Fig. 2, Fig. 3a). However, the expression level of these genes on methanol remained much higher than *pmxABC*. The expression level of the first two genes in the *pmxABC* operon, *pmxA* and *pmxB*, did not change significantly, while the expression of *pmxC* was downregulated on methanol (Fig. 3b). In summary, these results clearly indicated that the *pmo* genes play an important role in methane metabolism, and that methane may be a key enhancer for the expression of the *pmo* operon. The existence of a non-canonical form of ammonia/methane monooxygenase, *pmxABC,* was found in the genome of *Methylomonas* sp. DH-1. However, expression of *pmx* was very low, which suggested that the protein products of this operon may not be actively involved in the catalytic processes of *Methylomonas* sp. DH-1 when grown on methane or methanol.

Pyrroloquinoline quinone (PQQ)-dependent methanol dehydrogenase (*mxaFJGI*) and the PQQ biosynthesis gene cluster (*pqqABCDE*) were identified in the *Methylomonas* sp. DH-1 genome. The *mxaFJGI* operon for methanol oxidation (encoding two subunits of methanol dehydrogenase, cytochrome, and a protein of unknown function [*mxaJ*]) showed relatively high expression levels. While the expression of *mxaFJGI* actually decreased on methanol, the fold change was less than 2 (Fig. 2, Fig. 3c, Additional file 2: Table S2). The genes downstream of the *mxaFJGI* operon (which are chaperones, regulators, or have Ca^2+^ insertion functions) showed an intermediate or low level of expression that was 14- to 55-fold lower in comparison to the first four genes in the methanol dehydrogenase (MDH) operon (Additional file 2: Table S2). The PQQ biosynthesis gene cluster (*pqqABCDE*) encodes an essential system that provides cofactors for methanol oxidation. The expression level of this gene cluster was high (Additional file 2: Table S2). A homolog of *mxaF*, known as *xoxF*, was found in *Methylomonas* sp. DH-1. This gene showed a high expression level (Fig. 3c and Additional file 2: Table S2) when grown on methane, but was much lower than the expression of *mxaF*. Similar to the *pmo* operon, the expression of *xoxF* was significantly downregulated (3.65-fold change) on methanol (Fig. 3c). The homolog *xoxF* was firstly reported as part of a regulatory complex essential for the expression of methanol dehydrogenase in *Methylobacterium extorquens* AM1 [26] and currently has been identified as a predominant methanol dehydrogenase in *M. buryatense* 5GB1 [27]. Thus, it is possible that a high expression level of *xoxF* may contribute to methanol oxidation in *Methylomonas* sp. DH-1. Furthermore, direct coupling mode, in which methanol oxidation supplies electrons for methane oxidation, is most suitable mode of methane oxidation in gamma-proteobacterial methanotrophic bacteria [17]. Surprisingly, *xoxF* was highly downregulated during growth on methanol, similar to the pattern seen for *pmo*. Thus, we can postulate that *xoxF* may also play a role in methane oxidation in *Methylomonas* sp. DH-1 during growth on methane, and the exact contribution of *xoxF* in methane and methanol oxidation should be experimentally explored.

From the genomic analysis a group of genes in conventional type II methanotrophic metabolic pathways (such as the tetrahydromethanopterin (H_4_MPT) pathway and the tetrahydrofolate [H_4_F] pathway) were also identified in the genome of *Methylomonas* sp. DH-1. Previous studies have indicated that there are three possible pathways for formaldehyde oxidation, including H_4_MPT, H_4_F, and direct oxidation by formaldehyde dehydrogenase [28]. Genes for the direct oxidation of formaldehyde by formaldehyde dehydrogenase were not found in the *Methylomonas* sp. DH-1 genome. However, a broad-specificity aldehyde dehydrogenase (*ald*) was predicted from the genomic analysis, and the transcriptome analysis showed an intermediate expression level for this aldehyde dehydrogenase (Additional file 2: Table S2). Recently, the H_4_F pathway has drawn attention for its assimilatory function to convert formate to methylene-H_4_F. This pathway also contributes to formaldehyde oxidation in *M. extorquens* AM1 and *M. trichosporium* OB3b [14, 29]. All of the genes involved in the H_4_F pathway in *Methylomonas* sp. DH-1 were expressed at an intermediate level (Additional file 2: Table S2). During growth on methanol, the expression levels of genes in the H_4_F pathway were slightly increased compared to growth on methane (Fig. 3d). In particular, methenyltetrahydromethanopterin cyclohydrolase (*fch*) showed a fold change of 2.18 (Additional file 2: Table S2)*.* Among the three different formaldehyde oxidation processes, the H_4_MPT pathway serves as the key pathway in the type II model methanotroph, *M. trichosporium* OB3b [14]. Formaldehyde-activating enzyme (*fae*), which condenses formaldehyde with tetrahydromethanopterin (H_4_MPT) to produce methylene-H_4_MPT, is the first enzyme of the formaldehyde detoxification pathway through H_4_MPT. Interestingly, *Methylomonas* sp. DH-1 has three homologs of *fae* at different genomic locations. The orthologs *fae1* and *fae3* appear to be expressed at very low and at high levels on methane-grown cultures, respectively (Additional file 2: Table S2). However, the ortholog *fae2* was expressed at a very high level (10-fold higher than *fae*3) (Fig. 1 and Additional file 2: Table S2). The rest of the genes encoding key enzymes in the H_4_MPT pathway were also expressed at a high level on methane (Fig. 1 and Additional file 2: Table S2). Thus, our transcriptomic data analysis revealed that the genes in the H_4_MPT pathway showed high expression at the transcriptional level, indicating this pathway was likely key for formaldehyde oxidation in *Methylomonas* sp. DH-1. Interestingly, the expression of *fae2* was upregulated on methanol compared to methane (Fig. 2, Fig. 3d), meaning formaldehyde oxidation via H_4_MPT was more active during growth on methanol. Most methanotrophs use a NAD-dependent formate dehydrogenase to oxidize formate to CO_2_ [30]. It has been reported that most of the reducing power for methane metabolism was provided by formate oxidation to CO_2_ [31]. *Methylomonas* sp. DH-1 has the formate dehydrogenase (*fdsABGCD)* gene cluster, encoding a NAD-dependent formate dehydrogenase and an additional single copy of the alpha subunit (*fdhA*). Most of the genes in the operon *fdsABGCD* were expressed at a high level, and no significantly different expression of *fdsABGCD* was observed at the transcription level between methane and methanol (Fig. 3d and Additional file 2: Table S2). Overall, the transcriptomic analysis illustrate that the H_4_MPT pathway may serve as the key pathway for formaldehyde oxidation in *Methylomonas* sp. DH-1, as the pathway genes were highly expressed. Similarly, activation of H_4_F and the H_4_MPT pathway in *M. buryatense* strain 5GB1 and *M. alcaliphilum* 20Z^R^ under methane growth were also observed [17, 18].

A complete set of functional genes for formaldehyde fixation through the ribulose monophosphate (RuMP) cycle, Embden-Meyerhof Parnas (EMP) pathway, the Entner-Doudoroff (ED) pathway, and the pentose phosphate (PP) pathway was also identified in the *Methylomonas* sp. DH-1 genome. More interestingly, the complete gene set implementing the serine cycle without the interconnected ethylmalonyl-CoA (EMC) cycle and TCA cycle existed in the genome of *Methylomonas* sp. DH-1. A transcriptional analysis of these key formaldehyde assimilation pathways is described below.

### Gene expression of C1 assimilation pathway under methane growth and its response to substrate shifts

**Fig. 4:**
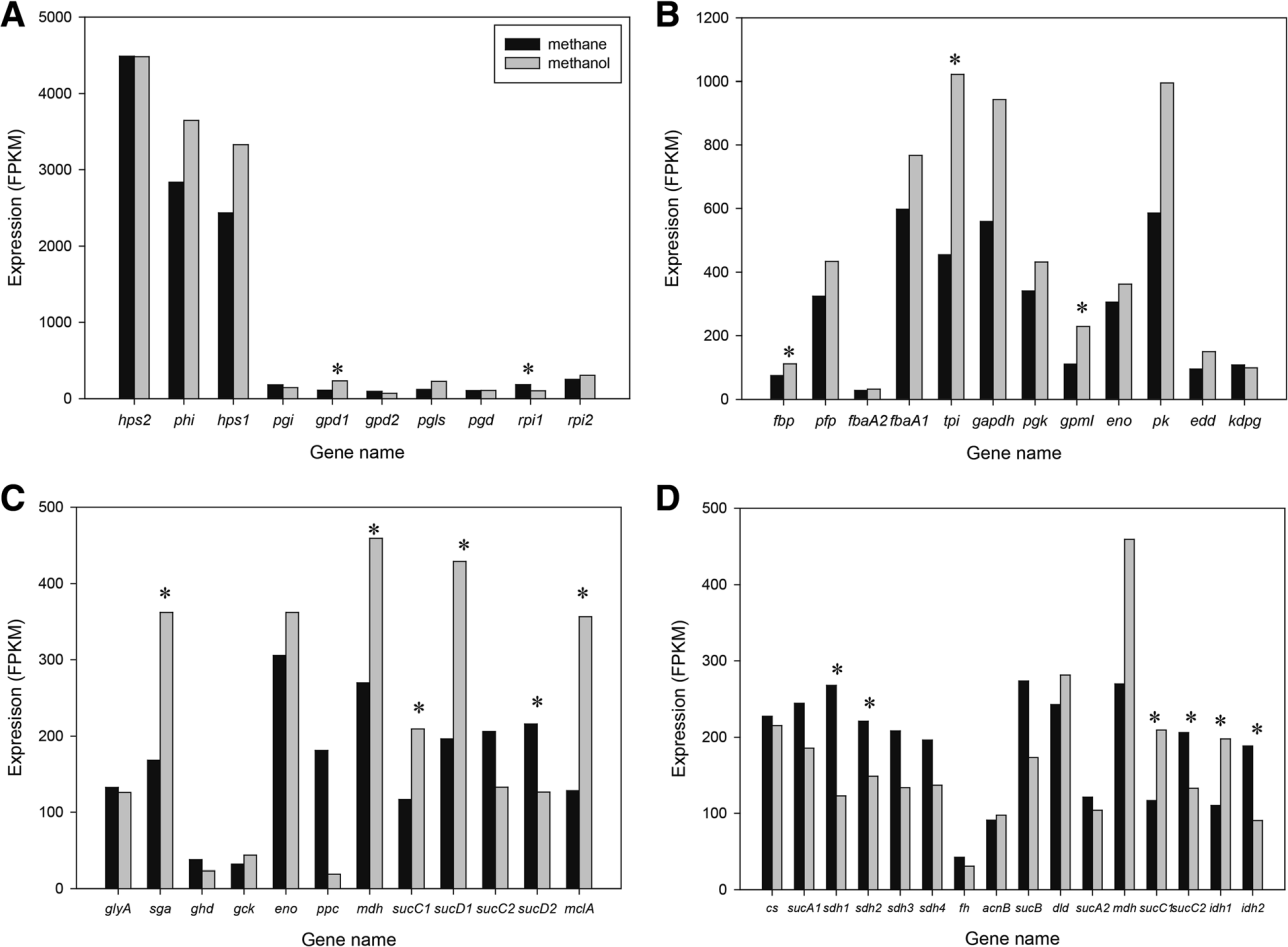
Differential expression of genes involved in C1 metabolism: **a** RuMP cycle, **b** ED and EMP pathways, **c** serine cycle, **d** TCA cycle in *Methylomonas* sp. DH-1 grown on methane (black) and methanol (gray). * Significantly different expression of genes between methane- and methanol-grown cultures (*P* < 0.05)

The RuMP pathway has been assumed to be the main pathway for C1 assimilation in the type I methanotrophs [30]. All genes for a complete RuMP cycle were identified in *Methylomonas* sp. DH-1, but those genes were transcribed at different levels (Fig. 1, Fig. 4a and Additional file 3: Table S3). Two key enzymes of the RuMP cycle are hexulose phosphate synthase (*hps*) and phophohexulo isomerase (*phi*). As expected, they were expressed at a very high level. In addition, another copy of hexulose phosphate synthase (AYM39_RS02745) was found in *Methylomonas* sp. DH-1, which was also expressed at a very high level. The expression of *hps* and *phi* did not change significantly between growth on methane and methanol (Fig. 2, Fig. 4a and Additional file 3: Table S3). This could be because those enzymes are regulated by concentration of formaldehyde [32]. The transcriptional expression of enzymes involved in the downstream portion of the RuMP cycle (from fructose-6-phosphate) was at an intermediate level (18- to 49-fold lower than *hpi* and *hps*). Interestingly, the abundance of transcripts encoding the Embden–Meyerhof–Parnas (EMP) pathway was 3 to 5-fold higher than that for Entner–Doudoroff (EDD) pathway enzymes (Fig. 1 and Fig. 4b, and Additional file 3: Table S3). Furthermore, a putative pyruvate kinase (*pk*) showed a very high expression level. It seemed that significant carbon flux could occur via the EMP pathway. Recently, ^13^C-labeling analysis have shown that the dominant pathway for generating pyruvate is the EMP pathway, which replenishes up to 75% of pyruvate in *Methylomicrobium alcaliphilum* 20Z and *Methylomonas* sp. LW13 [16]. In our previous study, comparative genomic and phylogenetic analysis was performed for 17 *Methylomonas* strains including *Methylomonas* sp. DH-1 and *Methylomonas* sp. LW13, and we found that *Methylomonas* sp. DH-1 and *Methylomonas* sp. LW13 showed high average nucleotide identity [19]. Along with the similar of gene expression profile as *M. alcaliphilum* 20Z and *Methylomonas* sp. LW13, a similar carbon isotopic distribution in pyruvate may exist and the EMP pathway likely serves as the main pathway for C1 assimilation in *Methylomonas* sp. DH-1.

In a comparison between the transcriptome profiles of methane- and methanol-grown cultures, the expression levels of most genes in the RuMP cycle did not change significantly, with these exceptions: glucose-6-phosphate dehydrogenase (*gpd1*), 6-phosphogluconolactonase (*pgls*), and ribose 5-phosphate isomerase (*rpi*). Upregulation of some genes in the EMP pathway, including fructose-bisphosphatase (*fbp*), triose-phosphate isomerase (*tpi*), and phosphoglycerate mutase (*gpml*), was observed. Transcription of pyruvate kinase was also upregulated on methanol compared to methane (Fig. 4a, b, and Additional file 3: Table S3).

### Targeted transcriptomic analysis of serine and TCA cycle suggested flux shifts under methanol-grown cultures

The genomic analysis suggested that *Methylomonas* sp. DH-1 possesses a complete serine cycle [19]. This is a typical C1 assimilation pathway of the type II methanotrophs, and is not a common feature in type I methanotrophs. All of the genes involved in the serine cycle were identified in the genome of *Methylomonas* sp. DH-1. Interestingly, but not surprisingly, those genes were expressed when grown on methane or methanol (Fig. 1, Fig. 4c, and Additional file 4: Table S4). Among them, D-glycerate dehydrogenase (*dgd*) and glycerate kinase (*gck*) were expressed at rather low expression levels; in contrast, phosphopyruvate hydratase (*eno*) and malate dehydrogenase (*mdh*) were highly expressed. It was reported that a partial serine cycle without phosphoenolpyruvate carboxylase (*ppc*) in *M. buryatense* 5G was predicted to have a minor flux during growth on methane by steady state ^13^C metabolic flux analysis [33] and in silico simulation [17]. Conversely, the existence and expression in *Methylomonas* sp. DH-1 of phosphoenolpyruvate carboxylase (*ppc*), a key enzyme in the serine cycle that serves as a major entry point for CO_2_ in alpha-proteobacterial methanotrophs [34], may provide the capability for CO_2_ fixation and malate production. Notably, the expression of *ppc* was significantly downregulated on methanol compared to methane. There are two types of *ppc* involved in a serine cycle: a “non-regulated” type where enzymatic activity is not controlled by intermediates of the TCA cycle or the glycolysis pathway, and a “regulated” type where the enzymatic activity of *ppc* is subject to control by various metabolic effectors [30, 35]. An ortholog analysis suggests that *ppc* of *Methylomonas* sp. DH-1 belongs to the regulated group (Additional file 5: Figure S1). Transcription of *ppc* decreased dramatically (9.6-fold) on methanol compared to methane, possibly because *ppc* was subject to metabolic effectors that are better produced on methane. As noted above, the H_4_F pathway can function as a part of an assimilatory pathway (via serine) that converts formate to methylene H_4_F. In accordance with this, upregulation of the H_4_F pathway was observed in the transcriptome data for growth on methanol, and this in turn may have affected expression of the serine cycle (Fig. 2 and Additional file 4: Table S4). Replenishment of glyoxylate is an essential function of the serine cycle [30, 34, 36]. However, no homolog of isocitrate lyase or malate synthase in the glyoxylate shunt was found in *Methylomonas* sp. DH-1, similar to other obligate methanotrophs [16, 17]. In addition, an ethylmalonyl-CoA (EMC) cycle was not identified. Thus, *Methylomonas* sp. DH-1 appears to have a complete serine cycle for carbon conversion to acetyl-CoA and for CO_2_ fixation. Among the genes in the serine cycle, the key genes such as serine-glyoxylate aminotransferase (*sga*) and *mdh* was significantly upregulated, with a 2.1-fold and 1,7-fold change, respectivelly (Fig. 2). Especially, the malyl-coA lyase (*mclA*), which directly produce acetyl-CoA via serine cycle, has been upregulated with a 2,78-fold change on methanol-grown cells. That suggested an increase flux towards the serine cycle to contribute acetyl-CoA production during growth on methanol.

Typically, type I and type X methanotrophs differ from type II methanotrophs because the former groups possess an incomplete tricarboxylic acid (TCA) cycle and do not have 2-oxoglutarate dehydrogenase enzyme activity [33, 37, 38]. It has been suggested that the main function of the TCA cycle in methanotrophs is to provide precursors for de novo biomass synthesis, as opposed to providing reducing power to the system [30]. However, a recent study using ^13^C tracer analysis demonstrated that a complete oxidative TCA cycle operates in *M. buryatense* [33]. Similar to *M. buryatense*, our genome analysis indicate that *Methylomonas* sp. DH-1 encodes all of the essential genes for the TCA cycle, and these genes are expressed on both methane and methanol (Fig. 1, Fig. 4d, Additional file 4: Table S4). Most genes in the TCA cycle were expressed at a high or intermediate level under methane growth, except for fumarate hydratase (*fh*), which was expressed at a low level. The 2-oxoglutarate dehydrogenase complex, which plays a key role in the TCA cycle, also was expressed at a high level. In order to confirm whether *Methylomonas* sp. DH-1 operates a complete TCA cycle, the ability to convert 2-oxoglutarate to succinyl-CoA or succinate needed to be tested. A succinate dehydrogenase mutant was generated to confirm any 2-oxoglutarate dehydrogenase activity. Interestingly, the mutant strain showed no difference in its growth rate compared to the wild-type strain, and succinate was accumulated in the media (data not shown). This observation supports the hypothesis that *Methylomonas* sp. DH-1 possesses a complete oxidative TCA cycle. This finding might be useful for future metabolic engineering of the TCA cycle in *Methylomonas* sp. DH-1 to produce relevant products. During growth on methanol, the expression of key genes in the TCA cycle was significantly downregulated such as succinate-coA ligase (*sucCD*), succinate dehydrogenase (*sdh*) and isocitrate dehydrogenase (*idh1*) (Fig. 2, Additional file 4: Table S4) which suggested the decreasing flux towards TCA cycle under methanol growth.

### Upregulation of carotenoid and hopanoid biosynthesis pathways under methanol growth

**Fig. 5:**
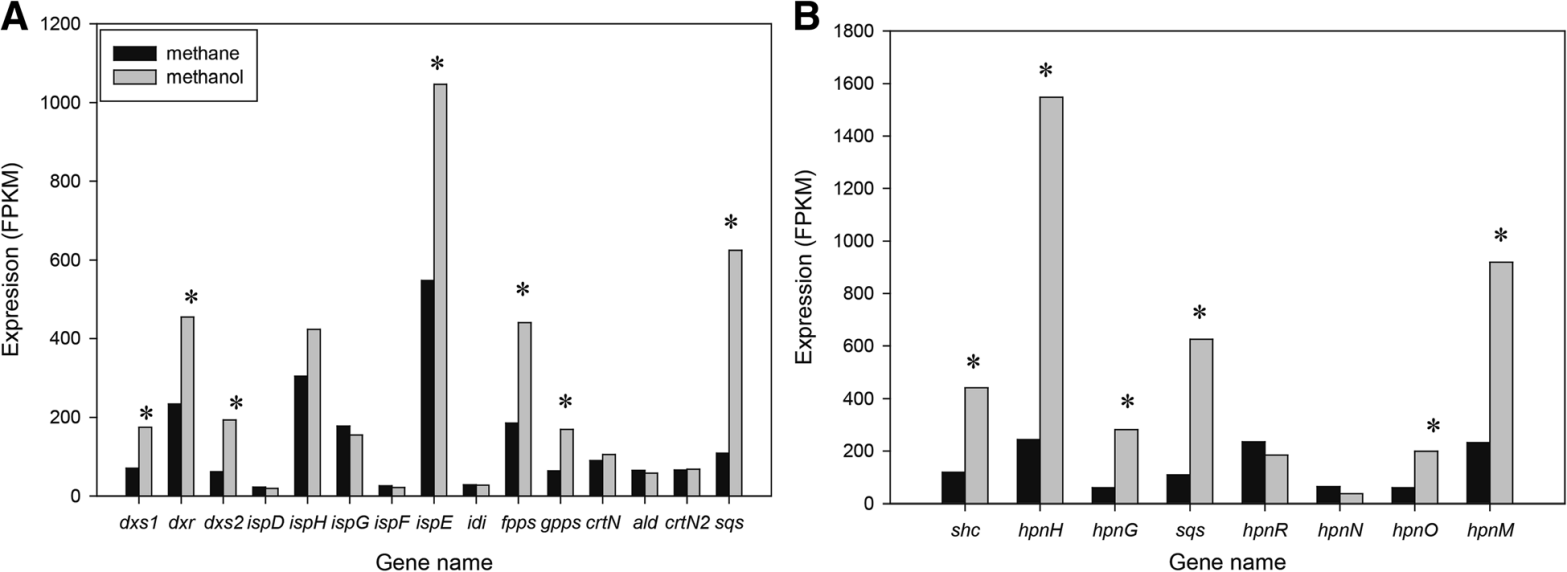
Differential expression of genes involved in secondary metabolites: **a** carotenoid and MEP pathways, **b** hopanoid biosynthesis pathway in *Methylomonas* sp. DH-1 grown on methane (black) and methanol (gray). * Significantly different expression of genes between methane- and methanol-grown cultures (*P* < 0.05)

The ability to produce various carotenoids demonstrates another potential value for *Methylomonas* sp. DH-1 in industrial use. *Methylomonas* sp. DH-1 carries a complete MEP pathway for carotenoid production, with two copies of 1-deoxy-D-xylulose-5-phosphate synthase (AYM39_RS06125 and AYM39_RS06125) [19]. Transcriptional profiling of *Methylomonas* sp. DH-1 grown on methane indicated that most of the genes in the MEP pathway were expressed at an intermediate or low level, with the exception of 4-hydroxy-3-methylbut-2-enyl diphosphate reductase (*ispH*), 4-(cytidine 5′-diphospho)-2-C-methyl-D-erythritol kinase (*ispE*), and 1-deoxy-D-xylulose-5-phosphate reductoisomerase (*dxr*), which were highly expressed (Fig. 5a and Additional file 6: Table S5). Among the genes in the MEP pathway, *ispE* showed the highest expression, and two homologs of *dxs* showed the lowest expression. Interestingly, a transcriptional comparison between the two carbon and energy sources indicated that many genes in the MEP pathway were significantly upregulated on methanol (Fig. 2). Among the upregulated genes in the MEP pathway, *dxs* showed particularly high upregulation on methanol: 2.5 and 3.1-fold changes were observed for *dxs1* and *dxs2*, respectively. Another important gene in the carotenoid synthesis pathway, the squalene/phytoene synthase gene (*sqs*), showed a low expression level on methane. Surprisingly, this gene was strongly upregulated on methanol, with a 5.7-fold change. On the other hand, the association of squalene, carotenoid and hopanoid biosynthesis response to stress in bacteria were reported [39, 40]. Thus, we can postulate that methanol might serve as a stress factor that induces the expression of genes in the MEP pathway. Consistent with the transcriptome data, a carotenoid profiling analysis showed that carotenoid production increased on methanol in *Methylomonas* sp. DH-1 (data not shown).

The carotenoid biosynthetic pathways share an upstream pathway of hopanoid biosynthesis [41]. Hopanoids are a group of natural pentacyclic compounds derived from the basic chemical structure of hopane. Studies in a range of bacteria suggest that hopanoids play a role in regulating membrane properties through an interaction with the outer leaflet of the outer membrane. From our genomic analysis, a complete hopanoid biosynthesis pathway is predicted in *Methylomonas* sp. DH-1. We predict the strain should therefore convert squalene to various hopanoids (including adenosylhopane, ribosylhopane, formylhopane, bacteriohopanetetrol), aminobacteriohopanetriol, and 3-methyl ABH) by hopanoid biosynthesis, using the associated radical S-adenosylmethionine (SAM) protein (*hpnH*), phosphorylase (*hpnG*), aminotransferase (*hpnO*), and hopanoid C-3 methylase (*hpnR*). Moreover, the existence of hopanoid biosynthesis-associated transporter (*hpnN)* and hopanoid biosynthesis protein (*hpnM*) in *Methylomonas* sp. DH-1 might enable the trafficking of hopanoids [42, 43]. Under methane growth, most of the genes in the hopanoid biosynthesis pathway were expressed at an intermediate level, except for *hpnH* and *hpnR*, which were expressed at a high level (Fig. 5b and Additional file 7: Table S6). Interestingly, when a carbon substrate was shifted from methane to methanol, the expression of hopanoid pathway genes increased significantly between 3.4-fold and 6.4-fold (Fig. 2, Fig. 5b, and Additional file 7: Table S6). Among them, *hphH* showed the highest upregulation, with a 6.4-fold change. It is tempting to speculate that such changes in gene expression in hopanoid biosynthesis pathway related to the ability to tolerate a high methanol concentration in DH-1 via modification of membrane properties.

### Transcriptional responses to oxidative stress during growth on methanol

**Fig. 6:**
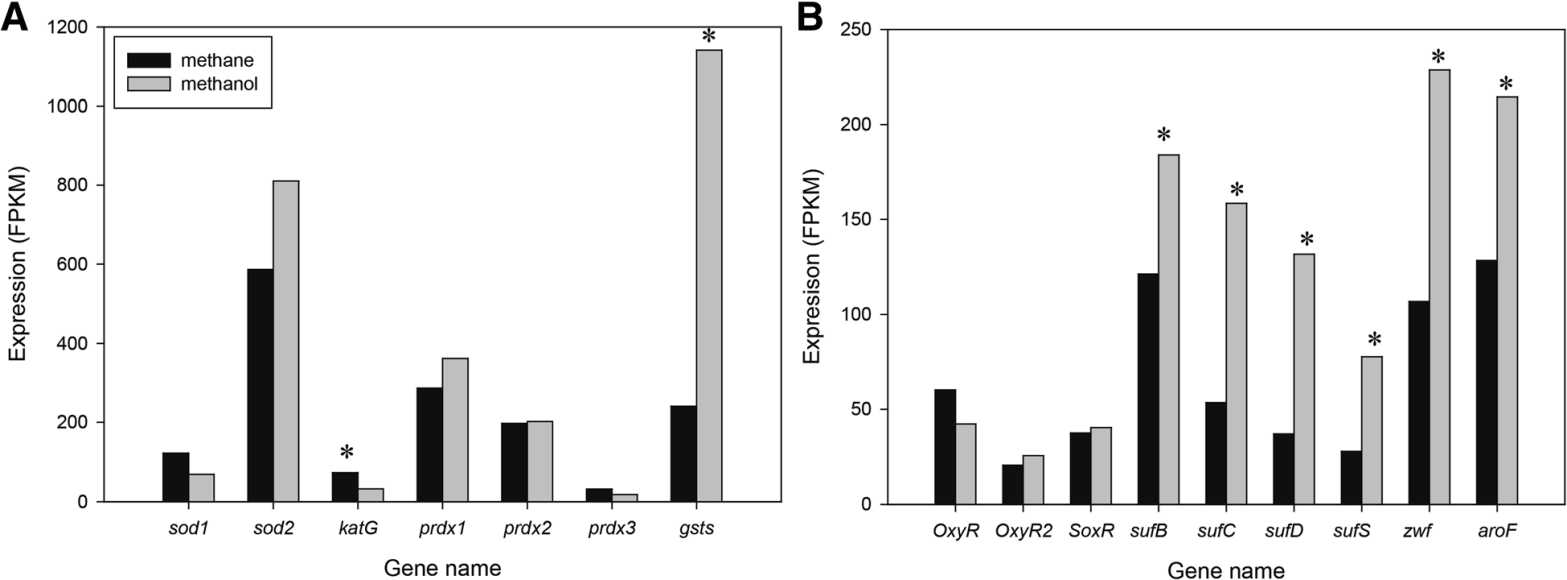
Differential expression of genes involved in the response to oxidative stress: **a** primary defense systems with scavenging enzymes, **b** *oxyR*, *oxyR2*, *soxR*, and their regulon genes in methane (black) and methanol (gray). * Significantly different expression of genes between methane- and methanol-grown cultures (*P* < 0.05)

As described above, methanol changed transcriptional levels for the carotenoid and hopanoid biosynthesis pathways, which both provide antioxidants [44]. In addition, a pigmented carotenoid in *Methylomonas* exhibited high antioxidative activities [45]. Based on the changes in the carotenoid and hopanoid profiles, we hypothesized that methanol might induce oxidative stress in *Methylomonas* sp. DH-1. Thus, we further attempted to determine transcriptional responses to oxidative stress during growth on methanol. Since CH4 biocatalysis is oxygen dependent, using oxygen-enriched air is a potential strategy for getting high-density growth of aerobic methanotrophs, for maximizing volumetric production of bacterial biomass, and for recombinant protein production. Thus, determining the effect of oxidative stress on the physiology and growth of methanotrophs is necessary. First we examined expression of antioxidant defense systems using scavenging enzymes, such as superoxide dismutase (*sod*), peroxiredoxin (*prdx*), and catalase (*cat*) (Fig. 6a and Additional file 8: Table S7). Both manganese superoxide dismutase and iron superoxide dismutase, which catalyze superoxide radicals into hydrogen peroxide and oxygen, were identified. Cu-Zn superoxide dismutase was not identified. A very high expression level of the gene encoding superoxide dismutase was observed in both of the treatments, but slightly upregulated in methanol. Catalases (*cat*) that decompose hydrogen peroxide to water and oxygen were also present in the *Methylomonas* sp. DH-1 genome. Expression of catalase in methane was intermediate, and it was slightly decreased in methanol. Three copies of *prdx* were identified, and all of them were expressed (but not significantly different) under the two conditions. There were six copies of glutathione S-transferase, which has an antioxidant role [46], and one of them (AYM39_RS19665) was strongly upregulated (2.2-fold change) in methanol (Fig. 6a, Additional file 8: Table S7). Under stressful conditions, however, these enzymes may be insufficient to protect cells from reactive oxygen species (ROS). Two other regulatory defense systems in gram-negative bacteria are induced under oxidative stress conditions: the *oxyR* system [47] responds to hydrogen peroxide, and the *soxR* and *soxS* systems respond to redox-active compounds [48]. Recently, a systems biology approach to decode the *oxyR*, *soxR*, and *soxS* regulatory networks under oxidative stress was reported in *E. coli* K12 MG1655 [49]. Since the regulators of oxidative stress in methanotrophs remain unclear, we performed an ortholog analysis between *E. coli* K12 MG1655 and *Methylomonas* sp. DH-1 using InParanoid [21] to compare the expression changes of regulators and their regulons during culture in methane and methanol. Because the genome annotation of *Methylomonas* sp. DH-1 still contains many gaps, an ortholog comparison of the DH-1 proteome and an accurate annotation of model strain *E. coli* K12 MG1655 was deemed a suitable approach for finding corresponding genes between the two strains. Based on the ortholog calculations, *oxyR* and *soxR*, but not *SoxS*, were identified in DH-1; these regulators were expressed at an intermediate level (Fig. 6b and Additional file 8: Table S7). Another copy of *oxyR* was identified, and it was expressed at a low level. In the case of *E. coli* K12 MG1655, expression levels of *oxyR* and *soxR* were upregulated under the oxidative stress treatment [49]. The expression levels of these regulators in DH-1 were not significantly changed in the methanol cultures. Thus, the regulatory defense system against oxidative stress might be different in methanotrophs compared to *E. coli*. A total of 68 genes in 51 transcription units (TUs) belong to the *oxyR*, *soxS*, and *soxR* regulons, which have been characterized [49]. Based on those results and our ortholog calculations, we further analyzed the expression of the *oxyR* and *soxRS* regulons in *Methylomonas* sp. DH-1. Thirty genes belonging to the *oxyR* and *soxRS* regulons exist in the DH-1 genome. Among them, 16 genes showed expression changes in methanol-grown cultures (Additional file 8: Table S7). Next, we analyzed the functions of those regulons. Among the 16 genes belonging to the *oxyR*, *soxR,* and *soxS* regulons, expression of glucose 6-phosphate dehydrogenase (*zwf*) was increased 2.1-fold in methanol. It has been reported that oxidative stress induces metabolic responses, such as the activation of *zwf* by SoxS, to increase NADPH pools and promote antioxidant defense by mediating the reduction of thioredoxins and glutaredoxins [50, 51]. Overexpression of amino acid biosynthesis genes as a means to overcome oxidative stress also has been reported [49]. The expression of 2-dehydro-3-deoxyphosphoheptonate aldolase (*aroF*), which promotes the synthesis of aromatic amino acids in *Methylomonas* sp. DH-1, increased possibly to overcome the shortage of essential amino acids. Other genes characterizing the cellular response to oxidative stress and damage repair, such as those involved in iron-sulfur (FeS) clusters, were highly overexpressed in methanol. Methanol likely induced oxidative stress in *Methylomonas* sp. DH-1 by activating a series of key enzymes in the damage repair and protection pathway, allowing cells to activate robust defenses against oxidative stress.

### High expression of endogenous plasmid genes plays a role in recombination

Endogenous plasmids are present in several methanotroph genomes [13, 19, 52]. The loss of native plasmid in *M. buryatense* 5G allowed the variant strain to receive small incompatibility group P (IncP) plasmids, which are broad host range vectors, via conjugation [52]. The native plasmid in *M. buryatense* 5G likely carries no genes essential for growth, since curing of that plasmid did not affect the growth phenotype [52]. *Methylomonas* sp. DH-1 contains an endogenous plasmid (pDH1) of 277 kb. In classifying the plasmid genes based on the clusters of orthologous groups (COGs) protein database, we found that replication, recombination, repair (category L), and transcription (category K) were overrepresented. Attempts to cure strains of this plasmid have failed (unpublished report) because it likely plays a significant role in the metabolism of DH-1. Conjugation to introduce *IncP*-based broad host range vectors (for expressing foreign genes) was not successful. This may have been due to the natural restriction-methylation barrier system that cleaves transforming plasmids before they can replicate in the cell. It is equally possible that incompatibility between foreign plasmid and endogenous plasmid caused stability and maintenance issues. The transcriptomic profile of *Methylomonas* sp. DH-1 showed that most of the genes in the pDH1 endogenous plasmid had very high or high expression levels (Table 1, Additional file 9: Table S8). Expression of the origin of replication in the plasmid (*DnaC*) was very high, in fact 10-fold higher than expression of the origin of replication in the main chromosome (*DnaA*). Although the exact copy number of the endogenous plasmid in DH-1 has not yet been quantified, the high expression of its origin of replication protein might reflect a high plasmid copy number. Thus, integrating foreign DNA cassettes into the endogenous plasmid may be an effective way to express heterologous genes in this strain. From a total of 242 protein-coding genes, 105 had significant expression changes. Among them, 46 genes and 59 genes were downregulated and upregulated in methanol-grown cultures, respectively (Additional file 9: Table S8).

## Discussion

In order to provide knowledge for methanotroph-based refineries, multi-omics can be used to define which metabolic pathways are active in certain conditions, and how cells response and adapt to new environments. In our previous work, the complete genome sequence of the newly isolated methanotroph *Methylomonas* sp. DH-1 was reported [19]. In the present study, a comprehensive characterization of the complete transcriptome of *Methylomonas* sp. DH-1 was provided and analyzed for the first time by an RNA-seq approach. This study provides in-depth knowledge about the metabolic pathways of this strain and reveals key differences in the transcriptional responses for certain metabolic pathways during growth in methane and methanol.

In the well-characterized methanotrophs, *pmo* is expressed at the highest level for cultures grown on methane [14–17]. A previous study has determined that transcripts of *pmoA* are very stable, with a half-life in the range of hours to days [53] which supported the hypothesis that the higher expression levels of *pmo* compared to other enzymes in the C1 oxidation pathway led to the first step of oxidizing methane is relatively slower compared to subsequent steps. During growth on methanol, the *pmo* operon was dramatically downregulated, likely because *pmo* genes are not involved in oxidizing methane. This is consistent with our previous study in which MMO activity has been dropped more than 3-fold when DH-1 growth on methanol [11]. Methane therefore may be a key regulator for the expression of the *pmo* operon. Similar to that of *M. trichosporium* OB3b, a type II model methanotroph, the expression of *pmo* and *smo* strongly affected by selection of different substrates [54]. The expression level of *pmo* and *smo* and their activity extremely dropped when the growth was shifted from methane to methanol [54].

*xoxF*, Ln^3+^ − dependent methanol dehydrogenase, has an important enzyme in methylotrophy, providing a new outlook on the distribution of methylotrophy in the bacterial community [55]. Interestingly, *xoxF* showed highly expression level without presence of Ln^3+^ and the similar expression pattern of *xoxF* and *pmo* supported the assumption that *xoxF* could contribute to the methane oxidation process in *Methylomonas* sp. DH-1. In agreement with our hypothesis, in most recent, the structure and function of *xoxF* in *M. buryatense* 5GB1C has been reported by investigating the possibility of interaction between pMMO and XoxF [56]. The results indicated a XoxF monomer may bind to pMMO and suggested an alternative structure of MDH-pMMO association. On the other hand, *M. trichosporium* OB3b showed very low expression level of *xoxF1* and *xoxF2* in methane and methanol [54]. Furthermore, the expression level of *xoxF1*, *xoxF2* as well as *mxaF* in *M. trichosporium* OB3b were decreased when grown on methanol with the presence of 10 μM copper, highlighting the differences in gene expression regulation in response to the type of carbon sources available between *Methylomonas* sp. DH-1 and *M. trichosporium* OB3b. It should be noted that while *M. trichosporium* OB3b exhibited the “copper-switch” to control the expression of alternative forms of methane monooxygenase, the “copper-switch” was not exist in *Methylomonas* sp. DH-1.

The discovery of typical type II methanotrophs metabolic pathways, such as the H_4_MPT pathway, H_4_F pathway, and complete serine cycle, in *Methylomonas* sp. DH-1 raised questions about the roles of these pathways in the central metabolism of this strain. From a previously published genome-scale model of *M. buryatense* 5GB1, a minor carbon flux is predicted via the H_4_MPT and H_4_F pathways [17, 18, 57]. However, these pathways were more active during growth on methanol, suggesting the improvement of carbon flux towards these pathway. This observation supports our hypothesis that the H_4_MPT and H_4_F pathways are mainly responsible for formaldehyde oxidation and contribute to carbon conversion via the serine cycle when grown on methanol.

A partial serine cycle without *ppc* has been determined in various type I methanotrophs such as *M. buryatense* 5GB1 and *M. alcaliphilum* 20Z^R^ which contributed a minor flux during growth in methane [17, 18]. Likewise, the complete gene set implementing the serine cycle in *Methylomonas* sp. DH-1 should allow the minor carbon flux needed to produce acetyl-coA. In the type II methanotroph *M. trichosporium* OB3b, which typically uses the serine cycle as a main pathway for C1 assimilation, there are two kinds of *ppc* gene: *ppc1* belongs to the non-regulated group and *ppc2* belongs to the regulated group [14]. The existence of two functionally identical but different regulation systems in *M. trichosporium* OB3b allows control of flux through phosphoenolpyruvate-oxaloacetate in response to the serine cycle, and this flux is never blocked completely [14]. The presence of only regulated *ppc* in *Methylomonas* sp. DH-1 indicates that carbon flux through the serine cycle can be blocked in the absence of effectors. During culture on methanol, expression of *ppc* was strongly downregulated, possibly because metabolite effectors which activate *ppc* expression were absent. The growth rate of *Methylomonas* sp. DH-1 in methanol was significantly decreased, perhaps because carbon flux via the serine cycle may have been blocked under methanol growth. However, most of the genes in the serine cycle were upregulated in methanol, suggesting significant shifts occur in C1 assimilation pathways, from RuMP to serine cycle. Along with RuMP cycle, the serine cycle also could take the role of producing acetyl-coA. EMP is main variant of RuMP pathway which play major role for C1 assimilation to produce NADH and ATP in type I methanotrophs [16–18]. The shifts decrease flux towards EMP pathway which subsequently decrease ATP production. Instead, the available electrons from methanol oxidation, which not used for methane oxidation under methanol growth, are transferred to the electron transport chain follow by producing ATP via oxidative phosphorylation. In order to determine the detailed rearrangement of metabolic network involved methanol-grown, ^13^C tracer analysis and constraint-based analysis of genome-scale metabolic network studies are needed. Thus, even the exist of the complete serine cycle in *Methylomonas* sp. DH-1 could not be main pathway for C1 assimilation, it could contribute to the control of carbon flux when shifting carbon substrates.

One unsolved question surrounding the central metabolism of type I methanotrophs is whether the oxidative TCA cycle is complete. In the recent time, a complete oxidative TCA cycle has been demonstrated to operate in *M. buryatense* 5GB1, and it has showed three separate pathways for converting 2-oxoglutarate to succinyl-CoA [33]. In another study, highly branched TCA cycle at the 2-oxoglutarate node also has been reported in *M. alcaliphilum* 20Z^R^ [18]. In this study, we also suggested *Methylomonas* sp. DH-1 possesses an complete oxidative TCA cycle. However, genomic analysis indicated at 2-oxoglutarate node, *Methylomonas* sp. DH-1 possesses 2-oxoglutarate dehydrogenase complex only but not 2-oxoglutarate ferredoxin oxidoreductase, succinate semialdehyde dehydrogenase or 2-oxoglutarate decarboxylase. Thus, the presence of highly branched TCA cycle in DH-1 remains to be elucidate. In addition, it seems that carbon flux though TCA cycle was reduced on methanol growth and the critical function of TCA under methanol growth has changed. In methanol-grown cells, TCA cycle mostly provide precursors for de novo synthesis but not reducing power such as NADH. Instead, it appears that the activation of formaldehyde oxidation in methanol growth could produce NADH.

In our previous study, the carotenoid biosynthesis pathways which derived from MEP pathway has been proposed [19]. The *dxs* is the first and one of the most important rate-limiting step in the MEP pathway, and overexpression of *dxs* could improve the production of several downstream secondary metabolites such as isoprenoid and carotenoid [58–61]. The flux shift occurred to MEP pathway via the strong upregulation of two *dxs* homologs (*dxs1* and *dxs2*) led to the accumulation of carotenoids in methanol-grown cultures. Meanwhile, the extremely upregulation of hopanoid biosynthesis pathway might related to membrane modifications under methanol growth (Fig. 2). The function of hopanoids has been characterized in several organisms, including methylotrophic bacteria [62, 63]. A lack of hopanoid biosynthesis increases sensitivity against toxins and osmotic stress. During growth on single-carbon compounds, methanol is generally converted to formaldehyde in the periplasm, and the formaldehyde is then transported and utilized in the cytoplasm. Given the toxic intermediates in this process, elevated maintenance of the inner and outer membranes is necessary. The role of hopanoids in maintaining membrane robustness and membrane barrier function is likely conserved across bacterial lineages. This function is possibly mediated through an interaction with lipid A in the outer membrane of *Methylobacterium extorquens* DM4 [63]. In addition, membrane function in the hopanoid-free *Methylobacterium extorquens* PA1 was lower [62]. Further investigation on the function of hopanoid biosynthesis pathway in property membranes of *Methylomonas* sp. DH-1 is needed to solve the question if hopanoid biosynthesis pathway could enable resistance to high methanol concentrations in *Methylomonas* sp. DH-1. Under methanol growth, the upregulation of carotenoid biosynthesis pathway, which produced pigmented carotenoid as antioxidant, and many regulatory defense systems against oxidative stress via damage repair and protection systems have been observed. It is speculated that such changes of these gene expression were induced by methanol which might induces ROS in *Methylomonas* sp. DH-1. A high expression of MEP pathway genes and an accumulation of carotenoids under stress conditions also describe previously reported in *Haematococcus pluvialis* [64]. That such speculation must be more rigorously confirmed by apply a system biology approach to reconstruct genome-wide of *OxyR*, *SoxR*, and *SoxS* regulatory networks under oxidative stress condition in methanotrophs.

## Conclusions

In conclusion, we have presented genomic and transcriptomic analyses of an industrially promising obligate methanotroph, *Methylomonas* sp. DH-1. The strain was grown on methane and methanol to analyze the shift of metabolism affecting by selection of substrates (Figs. 1, 2). While some metabolic functions had been reported in previous studies, several novel functions were identified and characterized in this strain. *Methylomonas* sp. DH-1 possesses the active EMP pathway which main route for C1 assimilation in this strain. In addition, *Methylomonas* sp. DH-1 also operates a complete oxidative TCA cycle. Along with the existence complete serine cycle, these pathways may function in C1 assimilation and energy production. We also identified a flux shift of metabolism towards formaldehyde oxidation pathway, serine and TCA cycle in *Methylomonas* sp. DH-1 when substrate was changed from methane and methanol. Furthermore, a significant upregulation of carotenoid and hopanoid biosynthesis pathways under methanol growth might explain the resistance to high methanol concentrations observed in *Methylomonas* sp. DH-1. It appears that methanotrophs are very dynamic to respond to change of environmental parameters.

## Additional files

Additional file 1: **Table S1.** Ten genes most highly expressed in *Methylomonas* sp. DH-1 in methane. (XLSX 12 kb)

Additional file 2: **Table S2.** Differential expression of genes involved in methane, methanol and formaldehyde oxidation in methane and methanol. (XLSX 14 kb)

Additional file 3: **Table S3.** Differential expression of genes involved in RuMP cycle, EMP and EDD pathway in methane and methanol. (XLSX 14 kb)

Additional file 4: **Table S4.** Differential expression of genes involved in serine and TCA cycle in methane and methanol. (XLSX 13 kb)

Additional file 5: **Figure S1.** Phylogenetic tree of phosphoenolpyruvate carboxylases. Sequence identifiers follow by species label. Node in red indicated “non-regulated” type and node in green indicated “regulated” type of *ppc*. Sequences were aligned and tree was created with ClustalX2 2.1 and rendered with iTOL. (DOCX 146 kb)

Additional file 6: **Table S5.** Differential expression of genes involved in MEP pathway and carotenoid biosynthesis pathway in methane and methanol. (XLSX 12 kb)

Additional file 7: **Table S6.** Differential expression of genes involved in hopanoid biosynthesis pathway in methane and methanol. (XLSX 11 kb)

Additional file 8: **Table S7.** Differential expression of genes involved in responding to the oxidative stress in methane and methanol. (XLSX 17 kb)

Additional file 9: **Table S8.** Differential expression of genes in pDH1 plasmid in methane and methanol. (XLSX 30 kb)

## Abbreviations

BAM: Binary alignment/map
BHT: Bacteriohopanetetrol
C1: Single-carbon
CDS: Coding DNA sequence
COG: Clusters of orthologous genes
DMAPP: Dimethylallyl diphosphate
EDD: Entner–Doudoroff
EMC: Ethylmalonyl-CoA
EMP: Embden–Meyerhof–Parnas
FDR: False discovery rate
FeS: Iron-sulfur
FPKM: Fragments per kilobase of exon per million fragments
H_4_F: Tetrahydrofolate
H_4_MPT: Tetrahydromethanopterin
IPP: Isopentenyl diphosphate
MDH: Methanol dehydrogenase
MEP: 2-C-methyl-D-erythritol 4-phosphate
MVA: Mevalonatic acid
PP: Pentose phosphate
PQQ: Pyrroloquinoline quinone
ROS: Reactive oxygen species
RuMP: Ribulose monophosphate
SAM: S-adenosylmethionine
SAM: Sequence alignment/map
TAC: Tricarboxylic acid
TU: Transcription unit
