# Supplementary material for: A comparative transcriptome analysis of the novel obligate methanotroph Methylomonas sp. DH-1 reveals key differences in transcriptional responses in C1 and secondary metabolite pathways during growth on methane and methanol
Source: BMC Genomics. 2019 Feb 12;20:130. doi: 10.1186/s12864-019-5487-6 (PMC6373157; doi:10.1186/s12864-019-5487-6)
Supplement: Supplementary file 5 — Figure S1. Phylogenetic tree of phosphoenolpyruvate carboxylases. Sequence identifiers follow by species label. Node in red indicated “non-regulated” type and node in green indicated “regulated” type of ppc. Sequences were aligned and tree was created with ClustalX2 2.1 and rendered with iTOL. (DOCX 146 kb) [file 12864_2019_5487_MOESM5_ESM.docx]

**Figure S1:** Phylogenetic tree of phosphoenolpyruvate carboxylases. Sequence identifiers follow by species label. Node in red indicated “non-regulated” type and node in green indicated “regulated” type of *ppc*. Sequences were aligned and tree was created with ClustalX2 2.1 [1] and rendered with iTOL [2]

**
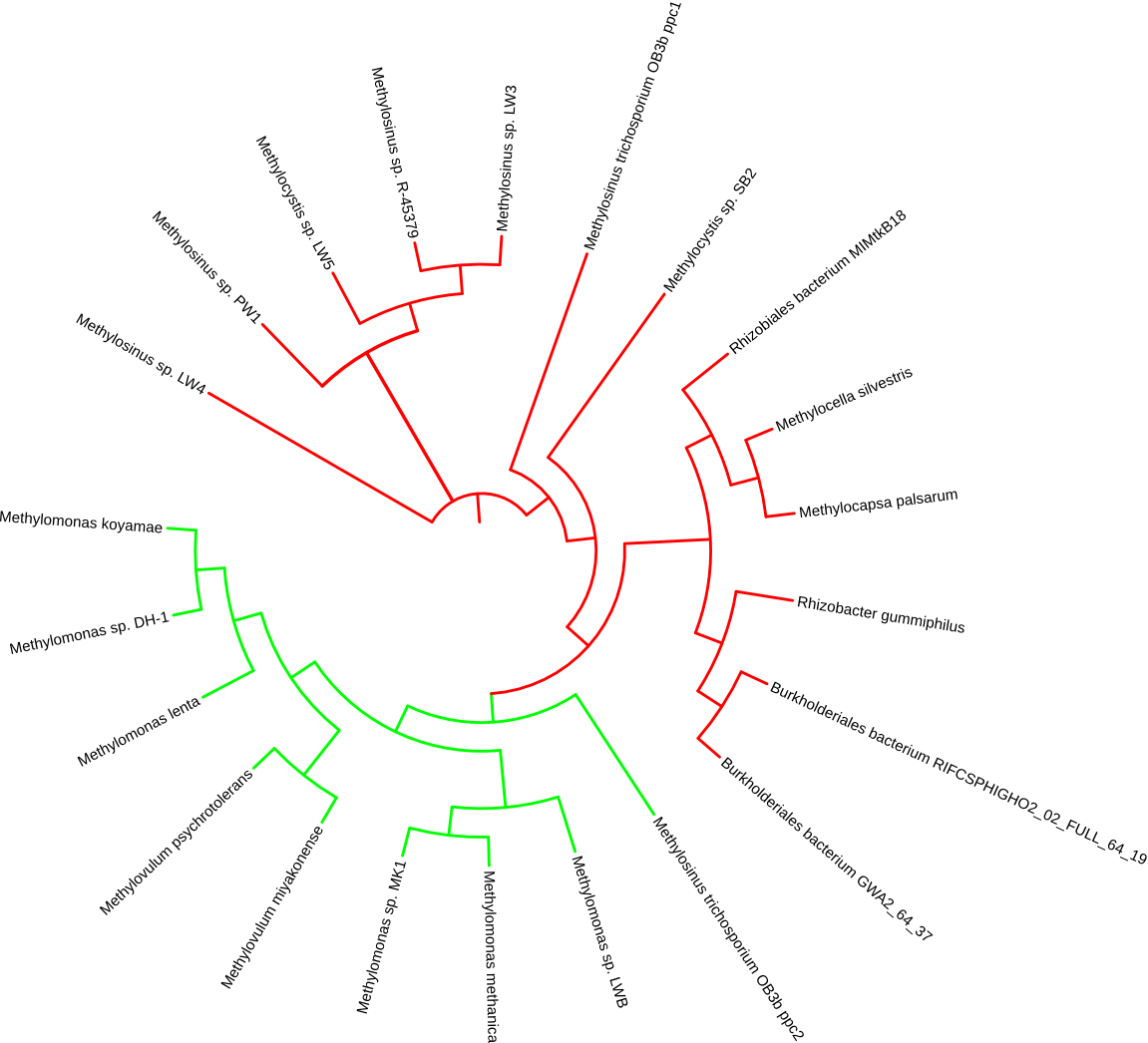
**

1. Larkin MA, Blackshields G, Brown NP, Chenna R, McGettigan PA, McWilliam H, et al. Clustal W and Clustal X version 2.0. Bioinformatics. 2007;23:2947-8
2. Letunic I and Bork P. Interactive Tree Of Life (iTOL): an online tool for phylogenetic tree display and annotation. Bioinformatics. 2007;23:127-8.
